# A Sleep Apnea Detection System Based on a One-Dimensional Deep Convolution Neural Network Model Using Single-Lead Electrocardiogram

**DOI:** 10.3390/s20154157

**Published:** 2020-07-26

**Authors:** Hung-Yu Chang, Cheng-Yu Yeh, Chung-Te Lee, Chun-Cheng Lin

**Affiliations:** 1 Heart Center, Cheng Hsin General Hospital, Taipei 112, Taiwan; amadeus0814@yahoo.com.tw; 2 Faculty of Medicine, School of Medicine, National Yang Ming University, Taipei 112, Taiwan; 3 Department of Electrical Engineering, National Chin-Yi University of Technology, Taichung 41170, Taiwan; cy.yeh@ncut.edu.tw (C.-Y.Y.); chunghengnice1@gmail.com (C.-T.L.)

**Keywords:** obstructive sleep apnea, single-lead electrocardiogram, deep learning, convolutional neural network

## Abstract

Many works in recent years have been focused on developing a portable and less expensive system for diagnosing patients with obstructive sleep apnea (OSA), instead of using the inconvenient and expensive polysomnography (PSG). This study proposes a sleep apnea detection system based on a one-dimensional (1D) deep convolutional neural network (CNN) model using the single-lead 1D electrocardiogram (ECG) signals. The proposed CNN model consists of 10 identical CNN-based feature extraction layers, a flattened layer, 4 identical classification layers mainly composed of fully connected networks, and a softmax classification layer. Thirty-five released and thirty-five withheld ECG recordings from the MIT PhysioNet Apnea-ECG Database were applied to train the proposed CNN model and validate its accuracy for the detection of the apnea events. The results show that the proposed model achieves 87.9% accuracy, 92.0% specificity, and 81.1% sensitivity for per-minute apnea detection, and 97.1% accuracy, 100% specificity, and 95.7% sensitivity for per-recording classification. The proposed model improves the accuracy of sleep apnea detection in comparison with several feature-engineering-based and feature-learning-based approaches.

## 1. Introduction

Obstructive sleep apnea (OSA) is a common sleep disorder that can cause shortness of breath or cessation of breathing, and is characterized by repeated pharyngeal collapse causing partial or complete obstruction of the upper airway, affecting ventilation during sleep [1]. It results in insufficient air entering the lungs and decreased blood oxygen concentration. Because of the lack of oxygen in the brain, patients are more likely to wake up, and so their sleep is frequently interrupted. OSA is highly prevalent in patients with cardiovascular disease, and is associated with the incidence and morbidity of hypertension, coronary heart disease, arrhythmia, heart failure, and stroke [2]. Polysomnography (PSG) is considered to be the gold standard for diagnosing OSA. It has to be done overnight in a sleep laboratory or sleep center, and requires several sensors to record multiple sleep physiological signals, including electroencephalography (EEG), electrooculogram (EOG), electrocardiogram (ECG), electromyogram (EMG), SaO2 saturation, thoracic abdominal effort, nasal–oral airflow, blood pressure, heart rate, and leg movement [3,4]. PSG measures the apnea–hypopnea index (AHI), which is defined as the sum of apneas and hypopneas per hour of sleep and has been widely used for diagnosing patients with OSA. According to the AHI, the severity of OSA can be classified as none (AHI<5), mild (5≤AHI<15), moderate (15≤AHI<30), or severe (AHI≥30) [5].

However, as PSG is an inconvenient, time-consuming, and expensive examination system, many studies in recent years have focused on the development of a portable and less expensive OSA diagnostic system using fewer physiological signals including blood oxygen concentration, ECG, chest and abdominal respiratory signals, breathing sounds, and the combined signals [6]. Evidence supports that single-lead ECG-based detection methods provide the highest global classification by studied population ratio among the algorithms based on a single-source sensor [6]. Analysis of ECG waveforms [7,8,9] and ECG-derived heart rates [10,11,12,13,14] are commonly used to detect sleep disordered breathing, including apnea, and hypopnea events.

Most of the previous works focused on feature engineering which requires a specific feature extraction method to extract ECG features from the ECG waveforms, RR intervals, heart rate variability (HRV), and ECG-derived respiration (EDR) signals. For example, several studies introduced the wavelet decomposition to extract the features from ECG waveforms [7,8,9]. Nguyen et al. [10] calculated the recurrence quantification analysis statistics of HRV signals to capture the dynamic changes of the cardiorespiratory system during OSA. Atri and Mohebbi [11] extracted a set of features from the bispectrum of HRV and EDR to acquire their nonlinear and non-Gaussian properties. Sharma and Sharma [12] extracted 16 features using the Hermite approximation of QRS waveforms including the Hermite expansion coefficients and the approximation error energy, and two features from RR intervals including the mean value and standard deviation of RR intervals. Song et al. [13] proposed a feature extraction method based on a hidden Markov model, and a feature selection approach using leave-one-out cross-validation to remove trivial features. Varon et al. [14] extracted two features derived from the changes in the morphology of QRS complexes and from heart rate and EDR using the orthogonal subspace projections. These two features were further combined with two features from the parameters of HRV including the standard deviation and the serial correlation coefficient. The classification techniques used by the previous studies include neural networks [7,10,12], K-Nearest Neighbor (KNN) [12,13], support vector machines (SVM) [8,10,12,13,14], random under sampling boosting [9], least-square SVM [11,12,14], the hidden Markov model [13], and linear discriminant analysis (LDA) [13,14].

Recently, several studies have proposed neural networks to automatically learn features. Singh and Majumder [15] proposed a pre-trained two-dimensional (2D) AlexNet model based on a convolutional neural network (CNN) to extract the features from 2D time-frequency images of ECG signals, and a decision fusion method consisting of the KNN, SVM, LDA, and Ensemble classifiers to improve the sensitivity for detecting apnea events. Wang et al. [16] proposed a modified LeNet-5 CNN model to extract features from 1D ECG signals and RR intervals, and to classify the normal and apnea events. Li et al. [17] developed a deep neural network to learn features from RR intervals, which belongs to unsupervised learning that only requires unlabeled ECG signals, and designed two types of classifiers including SVM and artificial neural network (ANN) to detect the apnea events. 

All of the above-mentioned techniques have demonstrated their superiority in the detection of apnea events. However, this study aims to further simplify the complexity of the signal preprocessing for the design of the CNN model. This study therefore proposes a sleep detection system based on a 1D deep CNN model and the use of single-lead 1D ECG signals. Only 1D ECG signals served as the input. The detection of QRS complexes and the analysis of ECG-derived heart rates are not required in this study. The signal preprocessing only includes Butterworth bandpass filtering and z-score normalization. Because the proposed CNN model has the same dimension of 1D with the input ECG signals, the signal preprocessing does not need additional signal transformation.

The rest of this paper is organized as follows. Section 2 describes the ECG recordings obtained from the MIT PhysioNet Apnea-ECG database, and presents the proposed apnea detection system based on a 1D deep CNN model. Results are given in Section 3. A discussion of the study findings is provided in Section 4. Finally, Section 5 concludes this study.

## 2. Materials and Methods

### 2.1. Materials

The MIT PhysioNet Apnea-ECG database [18,19] was recruited in this study. This database consists of a released dataset of 35 recordings and a withheld dataset of 35 recordings, digitized with at sampling rate of 100 Hz and with 12-bit resolution. The recording length varies from 401 to 587 min. Each recording contains a single-lead ECG signal and a set of annotations. Each 1-min ECG signal is annotated as label N or A, where N and A denote the normal and apnea events, respectively. All apnea events are obstructive or mixed. The events of pure central apnea and Cheyne-Stoke respiration are not included in the database. If a 1-min ECG signal contains hypopneas which have intermittent decreases in respiratory flow of less than 50% and decreases in oxygen saturation of at least 4%, and is accompanied by compensating hyperventilation, it is also annotated as apnea. These ECG recordings are divided into Class A (Apnea), Class B (Borderline), and Class C (Control). Recordings in classes A and B include at least one hour with an apnea index of 10 or more, and of 5 or more, respectively. Moreover, recordings in classes A, B, and C contain apnea or hypopnea of at least 100 min, between 5 and 99 min, and fewer than 5 min during the recording, respectively. The released and withheld datasets each contain 20 recordings of Class A, 5 recordings of Class B, and 10 recordings of Class C.

The released dataset was used for training the proposed 1D deep CNN model, and the withheld dataset was used to validate the performance of the proposed model. A total of 34,213 1-min ECG signals were extracted from the released and withheld datasets in this study. The released dataset has 16,979 min, of which 10,322 and 6657 min are annotated as normal and apnea events, respectively. The withheld dataset has 17,234 min, of which 10,717 and 6517 min are annotated as normal and apnea events, respectively.

### 2.2. The Proposed Apnea Detection System Based on a 1D Deep CNN Model

**Figure 1:**
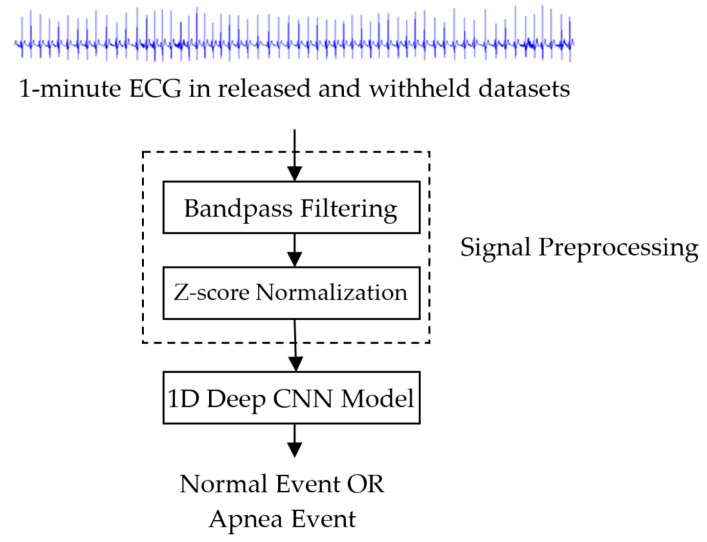
Block diagram of the proposed sleep apnea detection system based on a 1D deep convolutional neural network (CNN) model.

Figure 1 shows the block diagram of the proposed sleep apnea detection system based on a 1D deep CNN model. The input signal is a 1-min ECG signal with a length of 6000 samples in released and withheld sets. The signal preprocessing includes band-pass filtering and standardization. Each 1-min ECG signal was filtered through a fourth-order Butterworth bandpass filter with a 0.5 Hz to 15 Hz passband to reduce baseline drift and high-frequency interference. The Butterworth bandpass filter at 100 Hz sampling rate is implemented using the butter function from the Matlab signal processing toolbox [20]. Its difference equation is given as

(1) $$\begin{array}{ll} y\left(n\right) & =0.1242\;x\left(n\right)-0.2483\;x\left(n-2\right)+0.1242\;x\left(n-4\right)+\\ & 2.7422\;y\left(n-1\right)-2.7907\;y\left(n-2\right)+1.3311\times y\left(n-3\right)-0.2831\;y\left(n-4\right) \end{array}$$

**Figure 2:**
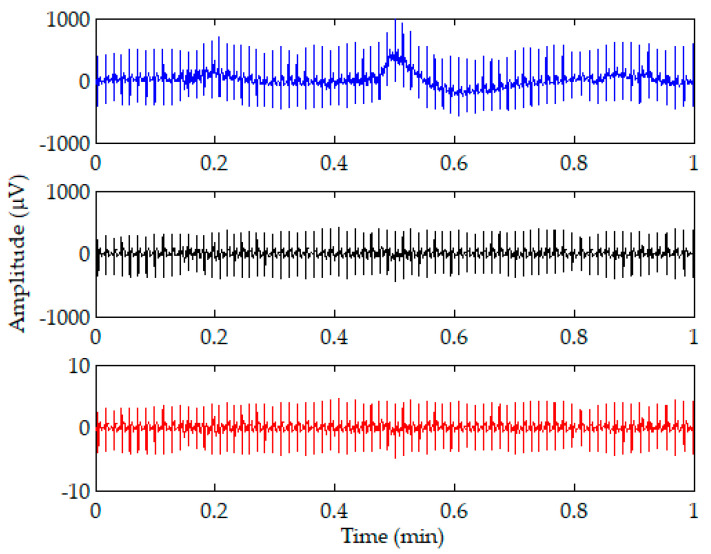
Illustration of a 1-min ECG signal (top) and the signals after bandpass filtering (middle) and z-score normalization (bottom).

The z-score function was used for standardizing the filtered ECG signals defined as follows:

(2) $$z=\frac{x-\mu }{\sigma }$$

where x is the input signal, and μ and σ are the mean and standard deviation of x, respectively. The z score shows how many standard deviations the input signal is from the mean. Figure 2 shows an illustration of the 1-min ECG signal (top) and the signals after bandpass filtering (middle) and z-score normalization (bottom). It is shown that most of the baseline drift can be removed after the bandpass filtering. The amplitudes of the filtered ECG signal ranging from −435 μV to 412 μV were reduced to amplitudes ranging from −4.8 μV to 4.6 μV after z-score normalization.

**Figure 3:**
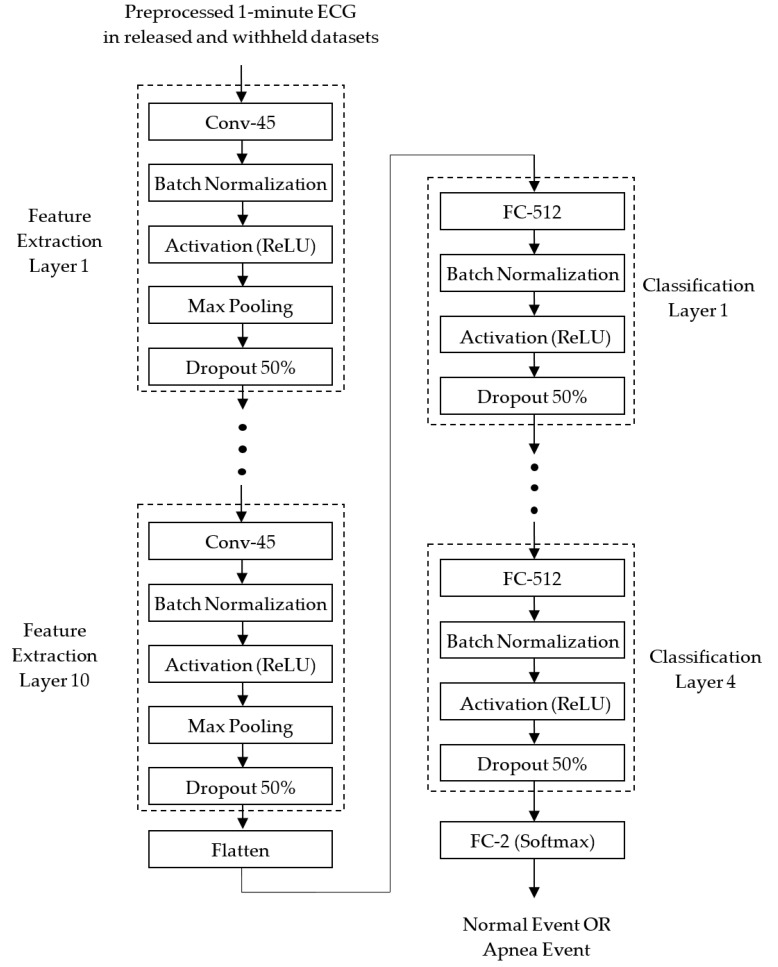
Block diagram of the proposed 1D deep CNN model for identifying normal and apnea events.

The preprocessed 1D ECG signals were then input into the proposed 1D deep CNN model for identifying normal and apnea events. Figure 3 depicts the block diagram of the proposed 1D deep CNN model. It is implemented using TensorFlow [21] and Keras [22]. TensorFlow is a machine learning framework, and also an open source software library that can support various algorithms for deep learning. Keras is a deep learning framework, and also a high-level application programming interface (API) which is capable of running on top of TensorFlow for building, training, and validating deep learning models of neural networks.

The proposed 1D deep CNN model consisted of feature extraction and classification stages. In the feature extraction stage, 10 identical feature extraction layers are designed to extract features from each 1-min ECG signal. Each feature extraction layer includes a 1D CNN layer (Conv-45) with 45 feature maps and a kernel length of 32, a batch normalization layer, an activation layer with the ReLU function, a max pooling layer with a pooling length of 2, and a dropout layer with a dropout rate of 0.5. After 10 feature extraction layers, a flattened layer is used to convert the 2D feature matrix consisting of 45 1D feature maps into a 1D feature vector, to be used by the classifier.

In the classification stages, 4 identical classification layers are designed to classify normal and apnea events based on the 1D feature vector. Each classification layer includes a fully connected (FC) layer with 512 neurons, a batch normalization layer, an activation layer with the ReLU function, and a dropout layer with a dropout rate of 0.5. After 4 classification layers, a softmax activation function is applied to calculate the probabilities of the two outputs of the FC-2 layer. The two outputs correspond to the normal and apnea events, respectively. The result of classification is the group corresponding to the output with a greater probability.

Both the CNN and FC layers use the He normal initialization method [23] to initialize the weights. The weights are initialized taking into account the size of the previous layer of neurons which helps to make the cost function reach the global minimum faster and more efficiently. The batch normalization layers added after the CNN and FC layers both in the feature extraction and classification layers are to normalize the data before entering the ReLU activation layer for improving the speed, performance, and stability of the neural network. The max pooling layers in the feature extraction layers are used to reduce the complexity of the network and the possibility of overfitting by selecting the maximum activation in the neighborhood of a neuron in a feature map. The use of the pooling size of 2 reduces the size of each feature map by a factor of 2, e.g., reducing the number of values in each feature map to one half the size. The dropout layers with a dropout rate of 0.5 are used to reduce overfitting by randomly omitting 50% of the nodes during the training process of the proposed CNN model. The overfitting would cause high training accuracy but low test accuracy. The proposed 1D deep CNN model was trained to minimize cross entropy using the Adam optimizer which is an extension of stochastic gradient descent, and computes individual adaptive learning rates for different parameters from estimates of the first and second moments of the gradients [24].

Table 1 shows the summary of the proposed CNN model. The input layer with the shape of (None, 6000, 1) is used to input a 1-min ECG signal. In each feature-extraction layer, the Conv-45 layer with 45 feature maps adopts a padding parameter of “same," so that each feature map has the same size as the input. Hence the output shape of the Conv-45 layer in the feature extraction layer 1 is (None, 6000, 45). The batch normalization and activation layers do not change the shape of the input. The max pooling layer with a pooling length of 2 and strides of 2 reduces the size of each feature map by half. Hence the output shape of the max pooling layer is reduced to (None, 3000, 45). Although the dropout layer ignores 50% of the nodes, it does not change the shape of the input. Because each feature extraction layer halves the size of each feature map, the output shape after 10 identical feature extraction layers is reduced to (None, 5, 45). After the flattened layer, a 1D feature vector with 225 features is extracted to be used for the classifier. The output shape of the FC-512 layer with 512 neurons is (None, 512). None of the batch normalization, activation or dropout layers change the shape of the input; hence, after 4 classification layers, the output shape is still (None, 512). The final FC-2 layer with softmax function reduces the output shape to (None, 2). 

## 3. Results

### 3.1. Performance of the Proposed Apnea Detection System for Per-Minute and Per-Recording Analysis 

The MIT PhysioNet Apnea-ECG containing 70 ECG recordings was used to evaluate the performance of the proposed apnea detection system in order to compare with some previous specific studies which used the same database. The training/released dataset is completely independent of the validation/withheld dataset. The commonly used local performance parameters for the apnea detection system are defined as follows [25]:

(3) $$\mathrm{Accuracy}=\frac{\mathrm{TP}+\mathrm{TN}}{\mathrm{TP}+\mathrm{FP}+\mathrm{TN}+\mathrm{FN}}\times 100\%$$

(4) $$\mathrm{Sensitivity}\;(\%)=\frac{\mathrm{TP}}{\mathrm{TP}+\mathrm{FN}}\times 100\%$$

(5) $$\mathrm{Specificity}\;(\%)=\frac{\mathrm{TN}}{\mathrm{TN}+\mathrm{FP}}\times 100\%$$

where TP is the number of true positive events (apnea events predicted as apnea events), FP is the number of false positive events (normal events predicted as apnea events), TN is the number of true negative events (normal events predicted as normal events), and FN is the number of false negative events (apnea events predicted as normal events). This study further plotted the receiver operating characteristic (ROC) to show the apnea detection performance at different classification thresholds, and calculated the area under the ROC curve (AUC) to measure the global performance [26,27].

Because of the randomness from the weight initialization in the CNN and FC layers and the dropping out in the dropout layer, we repeated 10 training and validation experiments to evaluate the performance of the proposed apnea detection system. The proposed 1D deep CNN model was trained and validated for 50 epochs in each experiment using 1-min ECG signals in the released and withheld datasets, respectively. The batch size was 10. The proposed model included about 1.5 million parameters and required about 402.4 million Multiply–Addition operations, which is about 804.8 million FLOPs. The training and validation of the proposed model were performed on a desktop computer equipped with Windows 10 Professional, Intel(R) Core(TM) i7-9700 3 GHz CPU, 32 GB RAM, and a GeForce(R) RTX2080 Super(TM) 8G graphics card. The training and validation took 61 s for the first epoch including the weight initialization, and 57 s for the second to fifth epochs. The time required to complete an experiment was about 48 min. Only 0.3 ms was required for testing a 1-min ECG signal as a normal or an apnea event using the trained model.

**Figure 4:**
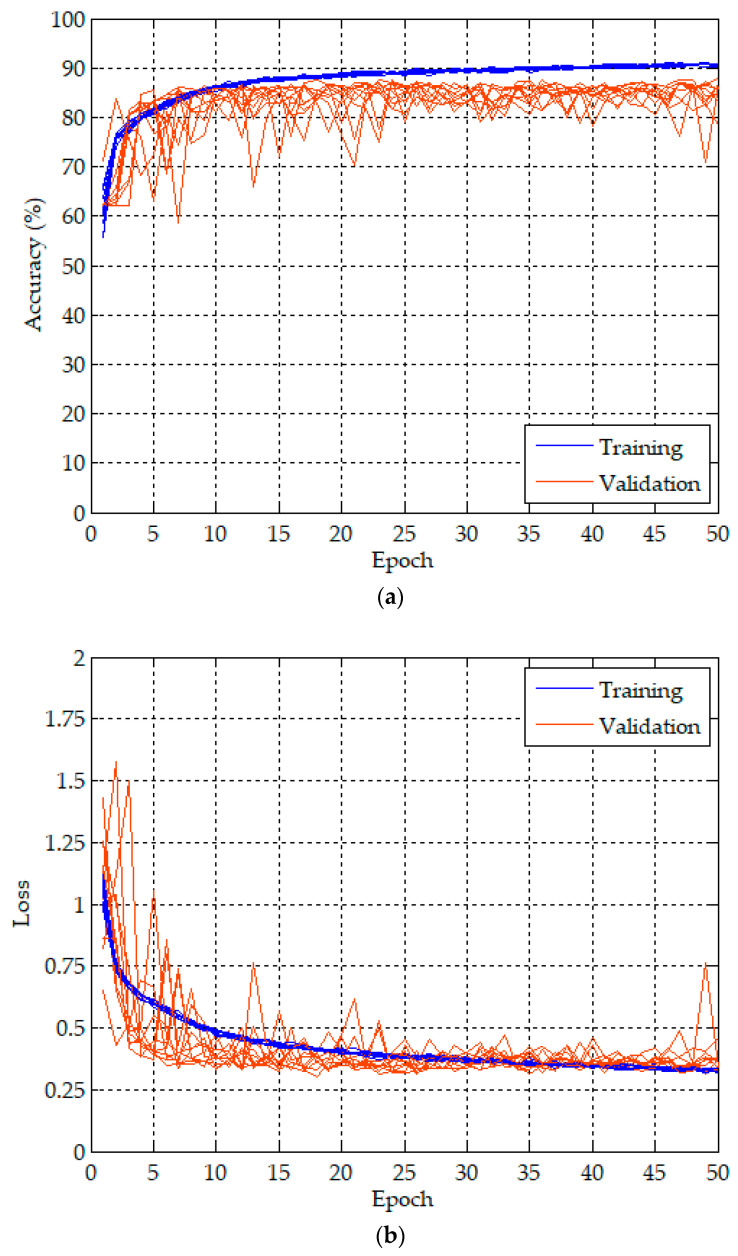
Training and validation history curves of per-minute apnea analysis for the proposed 1D deep CNN model: (**a**) accuracy curves, and (**b**) loss curves.

Figure 4 plots the training (blue lines) and validation (orange lines) history curves of the per-minute apnea detection of the proposed 1D deep CNN model for 10 experiments. Figure 4a,b are the accuracy and loss curves, respectively. The training accuracy and loss for an epoch are the average of the accuracies and losses over each batch of training data, respectively. Hence, we can observe a high degree of consistency from the training history curves of 10 experiments in both the accuracy and loss. However, the validation accuracy and loss for an epoch is computed using the entire validation data and the model, as it is the end of the epoch. The validation history curves of 10 experiments have a relatively larger degree of variation.

**Figure 5:**
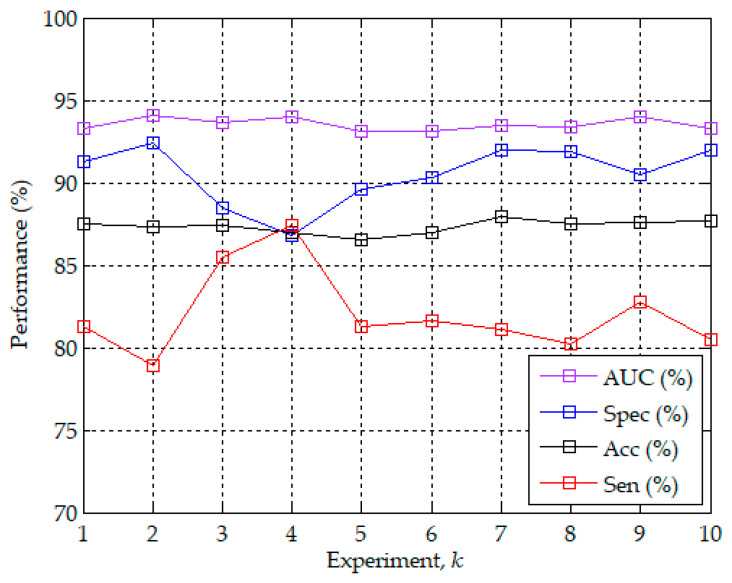
The best validation accuracy values of per-minute apnea analysis in each of the 10 experiments with the corresponding sensitivity, specificity, and the area under the (receiver operating characteristic (ROC)) curve (AUC).

**Figure 6:**
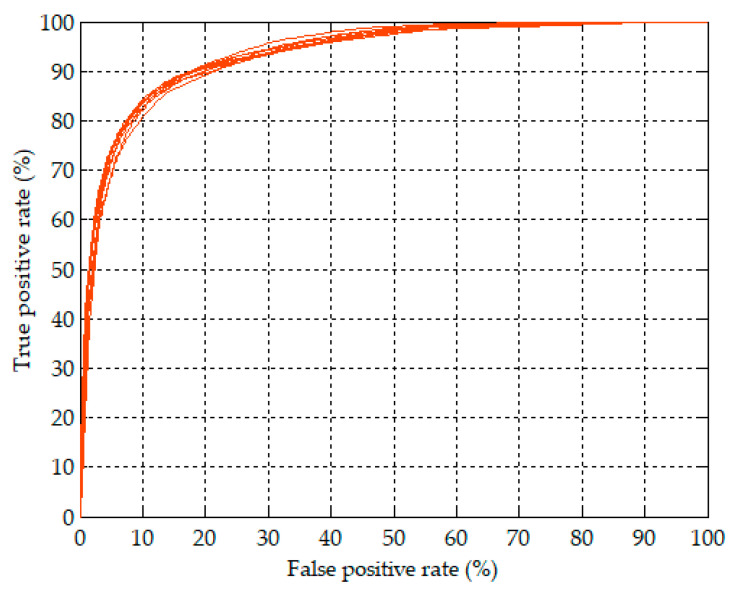
ROC curves corresponding to the model with the best validation accuracy values of per-minute apnea analysis in each of the 10 experiments.

Figure 5 further shows the best accuracy values of validation in each of the 10 experiments with the corresponding sensitivity, specificity and AUC. The best accuracy values were highly consistent and only ranged from 86.5% to 87.9%. The corresponding sensitivity, specificity, and AUC ranged from 78.9% to 87.4%, from 86.8% to 92.4%, and from 93.1% to 94.1%, respectively. The best validation accuracy of per-minute apnea detection among the 10 experiments was 87.9% of the seventh experiment with the corresponding sensitivity of 81.1%, specificity of 92.0%, and AUC of 93.5%. Figure 6 plots the ROC curves corresponding to the model with the best validation accuracy values of per-minute apnea detection in each of the 10 experiments. The 10 ROC curves showed a high degree of consistency of validation. Hence, except that the training results are highly reproducible in Figure 4, the best validation accuracy values in Figure 5 and the corresponding ROC curves in Figure 6 also demonstrate the high reproducibility of the validation results in the 10 experiments. Table 2 shows a summary of the confusion matrix and performance parameters of per-minute apnea detection for the training/released and validation/withheld datasets. The accuracy can reach 93.4% with sensitivity of 91.5% and specificity of 94.6% for the entire training/released dataset using the model with the best validation accuracy of 87.9%.

Based on the results of the per-minute apnea detection, we can further diagnose an ECG recording as a non-OSA subject or an OSA patient. According to the recommendation of the American Academy of Sleep Medicine (AASM), the OSA syndrome is defined as an AHI of five or greater [28]. The AHI value of each ECG recording is estimated by the results of per-minute apnea detection, defined as follows:

(6) $$\mathrm{AHI}=\frac{60}{L}\times N$$

where *L* denotes the number of 1-min ECG signals for a recording, *L*/60 is the number of hours for a recording, and *N* is the number of 1-min signals which are detected as apnea events. 

Table 3 summarizes the results of the per-recording analysis for the training/released and validation/withheld datasets. Both datasets have 35 ECG recordings. The recordings with AHI greater than or equal to five were diagnosed as OSA. The two datasets had the same diagnostic performance of per-recording analysis with accuracy of 97.1%, sensitivity of 95.7%, and specificity of 100%. Only one OSA patient was misdiagnosed as a non-OSA subject in both datasets. The correlation coefficients between the estimated AHI values of the proposed CNN model and the actual PSG AHI values provided by the MIT PhysioNet apnea-ECG database were 0.938 and 0.865 for the training/released and validation/withheld datasets, respectively.

### 3.2. The Effect of the Number of Feature Extraction Layers on the Performance of Apnea Detection

**Figure 7:**
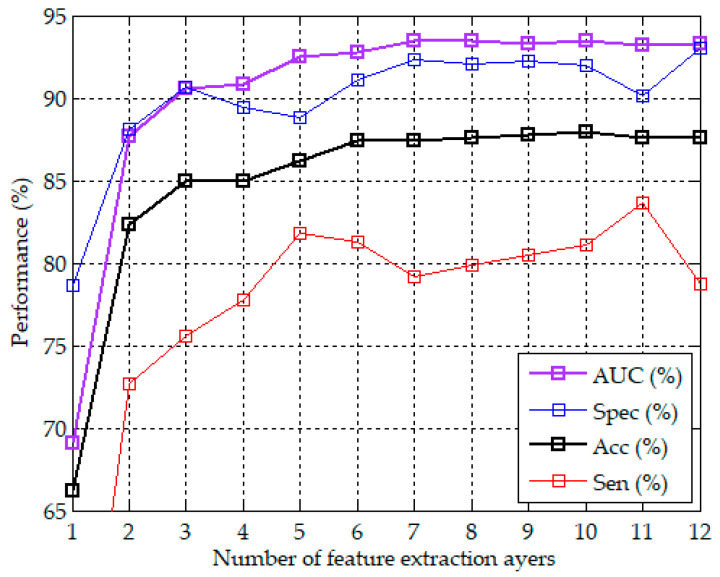
Curves of the number of feature extraction layers vs. the best accuracy in 10 experiments with the corresponding specificity, sensitivity, and AUC.

In addition to the apnea detection performance of the proposed CNN model using 10 feature extraction layers demonstrated above, we further analyzed the effect of the number of feature extraction layers on the performance. We evaluated the performance of the proposed CNN model using 1 to 12 feature extraction layers, respectively. Because each feature extraction layer included a max pooling layer with a pooling length of two, the more feature extraction layers, the fewer the extracted features. These CNN models used the same four FC-based classification layers. Each CNN model repeated 10 experiments. Figure 7 plots the curves of the number of feature extraction layers vs. the best accuracy in 10 experiments with the corresponding specificity, sensitivity, and AUC. It can be observed that the model using only six feature extraction layers achieved the accuracy of 87.4% with the corresponding specificity of 91.1%, sensitivity of 81.3%, and AUC of 92.8%. The accuracy and the corresponding AUC slightly increased with the number of feature extraction layers from 6 to 10 layers, and slightly decreased after 10 layers. The specificity and sensitivity did not increase with the layers. A higher specificity was accompanied by a lower sensitivity, and vice versa. The proposed CNN model with 10 feature extraction layers had the best accuracy of 87.9% among the models using 1 to 12 feature extraction layers.

## 4. Discussion

The study has demonstrated that the proposed apnea detection system based on a 1D deep CNN model can extract the features from 1-min ECG signals and classify them into normal and apnea events. The released and withheld datasets each with 35 ECG recordings from the MIT PhysioNet apnea-ECG database were used to train and validate the proposed system. The input signals are the 1D ECG signals. The signal preprocessing only needs the Butterworth band-pass filtering and z-score normalization. The proposed 1D deep CNN model includes 10 identical feature extraction layers, a flattened layer, 4 identical classification layers, and a softmax FC layer. A total of 10 1D-CNN layers were designed to extract the features of the 1-min ECG signal, and a total of 5 FC layers were designed to classify the normal and apnea events based on the extracted features. The feature extractor and classifier are trained together by the Adam optimizer [24]. 

Several studies have proposed apnea detection approaches based on the features extracted from the RR intervals or heart rates [10,11,12,13,14]. However, the calculation of the RR intervals or heart rates requires an accurate QRS detection algorithm. A wrong R peak would cause one or two wrong RR intervals which are difficult to correct, thus reducing the accuracy of the extracted features. In contrast, this study only used the original 1D ECG signals as the input, without the inclusion of RR intervals or heart rates. Neither the detection of the QRS complexes nor the analysis of RR intervals or heart rates were required in this study. Moreover, most of the previous work was focused on feature engineering which needs prior knowledge of OSA to extract features using a particular method [7,8,9,10,11,12,13,14]. This means that the feature engineering process needs a large amount of labor to determine the most representative features of apnea events. In this study, the feature extraction and classifier are designed and trained together, and the features can be automatically extracted by the proposed CNN model.

Table 4 compares the signal preprocessing methods of the proposed apnea system with three feature-learning-based and three feature-engineering-based systems. These studies all adopted the MIT PhysioNet apnea-ECG database, and the released and withheld datasets were used for training and validation, respectively. Our study and the studies of Singh and Majumder [15], Wang et al. [16], and Li et al. [17] proposed feature-learning-based methods which can automatically learn the features of ECG signals or RR intervals using neural networks. The studies of Sharma and Sharma [12], Song et al. [13], and Varon et al. [14] focused on feature-engineering-based methods. Only our study and the study of Singh and Majumder [15] did not require R-peaks detection and RR interval calculation. However, the apnea detection system proposed by Singh and Majumder [15] is based on a 2D CNN model, and thus a continuous wavelet transform is required to transfer the 1D ECG signals to 2D scalogram images in the signal preprocessing stage. In comparison with the 1D CNN model, the 2D CNN model needs more parameters and has higher computational costs. Because the proposed CNN model has the same dimension of 1D as the input ECG signals, the signal preprocessing does not require additional signal transformation in this study. The other studies all required R-peaks detection and RR interval calculation. The media filtering [13,16,17] and R-peaks correction [14] have often been used to eliminate the physiologically uninterpretable points. Interpolation [16,17] has been used to interpolate all RR interval series to have the same length. The QRS complex extraction and EDR derivation were also included in the studies of Sharma and Sharma [12], and Song et al. [13], and Varon et al. [14], respectively. Accordingly, in comparison with these studies [12,13,14,15,16,17], the proposed apnea detection system can simplify the complexity of the signal preprocessing, only including bandpass filtering and z-score normalization.

Table 5 compares the performance of the proposed 1D deep CNN model with the previous studies for the per-minute apnea detection. The sensitivity, specificity, accuracy, and AUC of all studies ranged from 79.5% to 90.0%, from 82.1% to 92.0%, from 83.8% to 87.9%, and from 0.83 to 0.95, respectively, for the per-minute apnea detection. The best accuracy is the 87.9% of the proposed method, followed by the 87.6% of Wang et al. [16], and the 86.2% of Singh and Majumder [15] and Song et al. [13]. The proposed method also has the best specificity of 92.0%, followed by the 90.3% of Wang et al. [16], and the 88.4% of Sharma and Sharma [12] and Song et al. [13]. Singh and Majumder [12] has the best sensitivity of 90.0%, but their specificity is only 83.8%. The sensitivity of Li et al. [17] is the second highest at 88.9%, which is only slightly lower than the 90% of Singh and Majumder [15], but their specificity is also only 82.1%. The best AUC is the 0.95 of Wang et al. [16], which is slightly higher than the 0.94 of the proposed method and of Song et al. [13]. The AUC values of other studies are all lower than 0.90.

Table 6 further compares the performance of the proposed 1D deep CNN model with the previous studies for the per-recording classification. The performance of per-recording classification of the study of Varon et al. [14] is not listed in Table 6. They divided 70 recordings into normal, borderline, and apnea classes. The AHI was computed for each recording. Their results showed that the normal subjects could be separated from apnea patients with an accuracy of 100% if AHI of 10 or greater was used to diagnose apnea patients. However, they did not provide the details of the borderline subjects. All of the diagnostic criteria shown in Table 6 were the estimated AHI of five or greater. The withheld dataset includes 35 recordings consisting of 23 OSA patients and 12 non-OSA subjects. The best accuracy is the 100% of Singh and Majumder [15] and Li et al. [17]. In our study, an OSA patient was misclassified as a non-OSA subject. Hence, the accuracy of the proposed approach is 97.1% with sensitivity of 95.7% and specificity of 100%. The studies of Sharma and Sharma [12] and Song et al. [13] have the same performance as our study. Their sensitivity of 95.8% should be corrected to 95.7%. In the study of Wang et al. [16], a non-OSA subject was misclassified as an OSA patient. Hence, their accuracy is 97.1%, with sensitivity of 100% and specificity of 91.7%.

The studies of Singh and Majumder [15] and Li et al. [17] did not provide the correlation coefficients between the estimated and actual PSG AHI values. The correlation coefficient of the proposed model is 0.865, which is slightly higher than the 0.860 of Song et al. [13] and the 0.841 of Sharma and Sharma [12]. It is worth noting that Wang et al. [16] had a much higher correlation coefficient of 0.943, but lower accuracy for the per-minute apnea detection in comparison with our study. This inconsistent result may be caused by the inconsistent definition of the correlation coefficient. If we calculate the correlation coefficient between the estimated AHI values and the AHI values according to per-minute annotations, it is 0.948 for the proposed model, which is slightly higher than the 0.943 of Wang et al. [16]. Hence, it is possible that the correlation coefficient in Wang et al. [16] is calculated between the estimated AHI values and the AHI values according to the annotations of the 1-min ECG signals, not between the estimated AHI values and the actual PSG AHI values.

The limitation of the study is the lower sensitivity of 81.1% for per-minute apnea detection in comparison with the other studies listed in Table 5. A lower sensitivity would cause more apnea events to be misidentified as normal events, and hence would lower the estimated AHI and may cause OSA patients to be misclassified as non-OSA subjects for the per-recording classification. Accordingly, our future work will focus on enhancing the sensitivity of the per-minute apnea detection for further increasing the feasibility of using a single-lead ECG to detect apnea events and diagnose OSA patients. 

## 5. Conclusions

This study proposes a 1D deep CNN model for the detection of apnea events only using 1D ECG signals as input. The proposed CNN model includes 10 CNN-based feature extraction layers and 4 FC-based classification layers. The signal preprocessing only needs Butterworth bandpass filtering and z-score normalization without the detection of QRS complexes, the analysis of RR intervals, or additional signal transformation. The proposed CNN model was trained and validated by the released and withheld datasets of the MIT PhysioNet Apnea-ECG database, respectively. In comparison with several previous studies, the proposed method has the best accuracy of 87.9% and specificity of 92.0% with corresponding sensitivity of 81.1% and AUC of 0.94 for per-minute apnea detection. The performance of per-recording classification can achieve the accuracy of 97.1% with sensitivity of 95.7% and specificity of 100%. The proposed system can be served as a convenient and advanced diagnosis system of OSA only using 1D ECG signals. If the estimated AHI is greater than or equal to five, it is recommended to follow up with a PSG test to confirm the severity of OSA.

## Figures and Tables

**Table 1:** Summary of the proposed 1D deep CNN model.

Layers	Parameters	Output Shape
Input		(None, 6000, 1)
Feature Extraction Layer 1		
Conv-45	filters = 45, kernel size = 32 padding = ‘same’ kernel initializer = ’he_normal’	(None, 6000, 45)
Batch Normalization		(None, 6000, 45)
Activation	ReLu	(None, 6000, 45)
Max Pooling	pool size = 2, strides = 2	(None, 3000, 45)
Dropout	dropout rate = 0.5	(None, 3000, 45)
● ● ●		
Feature Extraction Layer 10		
Conv-45	filters = 45, kernel size = 32 padding = ‘same’ kernel initializer = ’he_normal’	(None, 11, 45)
Batch Normalization		(None, 11, 45)
Activation	ReLu	(None, 11, 45)
Max Pooling	pool size = 2, strides = 2	(None, 5, 45)
Dropout	dropout rate = 0.5	(None, 5, 45)
Flatten		(None, 225)
Classification Layer 1		
FC-512	units = 512 kernel initializer = ’he_normal’	(None, 512)
Batch Normalization		(None, 512)
Activation	ReLu	(None, 512)
Dropout	dropout rate = 0.5	(None, 512)
● ● ●		
Classification Layer 4		
FC-512	units = 512 kernel initializer = ’he_normal’	(None, 512)
Batch Normalization		(None, 512)
Activation	ReLu	(None, 512)
Dropout	dropout rate = 0.5	(None, 512)
FC-2	Softmax	(None, 2)

**Table 2:** Summary of the confusion matrix and performance parameters of per-minute apnea analysis for the training/released and validation/withheld datasets.

Dataset		Predict	N	A	Sen (%)	Spe (%)	Acc (%)
	Label						
Training/Released	N		9760	562	91.5	94.6	93.4
	A		565	6092			
Validation/Withheld	N		9863	854	81.1	92.0	87.9
	A		1230	5287			

N: Normal event; A: Apnea event; Sen: Sensitivity; Spe: Specificity; and Acc: Accuracy.

**Table 3:** Summary of the results of the per-recording analysis for the training/released and validation/withheld datasets.

Dataset	Recordings	Diagnostic Criteria	Sen (%)	Spe (%)	Acc (%)	Corr.
Training/Released	35	5	95.7	100	97.1	0.938
Validation/Withheld	35	5	95.7	100	97.1	0.865

Sen: Sensitivity; Spe: Specificity; Acc: Accuracy; and Corr.: Correlation.

**Table 4:** Comparison of the signal preprocessing methods of the proposed apnea system with the previous studies.

Reference	Signal Preprocessing Methods
**Feature-Learning-Based Systems**	
Our Study	Bandpass Filtering + Z-score Normalization
Singh and Majumder [15]	Bandpass Filtering + Continuous Wavelet Transform + Zerocenter Normalization
Wang et al. [16]	FIR Filtering + R-peaks Detection + RR Interval Calculation + Median Filtering + Cubic Interpolation
Li et al. [17]	Bandpass Filtering + R-peaks Detection + RR Interval Calculation + Median Filtering + Interpolation
**Feature-Engineering-Based Systems**	
Sharma and Sharma [12]	Bandpass Filtering + R-peaks Detection + RR Interval Calculation + QRS Complex Extraction + Zero Padding
Song et al. [13]	Filter-Bank-Based R-peaks Detection + RR Interval Calculation + Median Filtering + EDR Derivation
Varon et al. [14]	Notch Filtering + DC Component Remove + Up Sampling + R-peaks Detection + R-peaks Correction + RR Interval Calculation + EDR Derivation

FIR: Finite impulse response; DC: Direct current; and EDR: ECG-derived respiration.

**Table 5:** Performance comparison of the proposed 1D deep CNN model with the previous studies for the per-minute apnea detection.

Reference	Methods	Sen (%)	Spe (%)	Acc (%)	AUC
**Feature-Learning-Based Methods**					
Our Study	The proposed 1D Deep CNN Model	81.1	92.0	87.9	0.94
Singh and Majumder [15]	Pre-trained AlexNet CNN + Decision Fusion	90.0	83.8	86.2	0.88
Wang et al. [16]	LeNet-5 CNN	83.1	90.3	87.6	0.95
Li et al. [17]	Auto-encoder + Decision Fusion	88.9	82.1	84.7	0.87
**Feature-Engineering-Based Methods**					
Sharma and Sharma [12]	Feature Engineering + LS-SVM	79.5	88.4	83.8	0.83
Song et al. [13]	Feature Engineering + HMM-SVM	82.6	88.4	86.2	0.94
Varon et al. [14]	Feature Engineering + LS-SVM	84.7	84.7	84.7	0.88

**Table 6:** Performance comparison of the proposed apnea detection system with the previous studies for the per-recording classification.

Reference	Recordings	Diagnostic Criteria	Sen (%)	Spe (%)	Acc (%)	Corr.
Our study	23 OSA Patients + 12 Healthy Subjects	5	95.7	100	97.1	0.865
Singh and Majumder [15]			100	100	100	-
Wang et al. [16]			100	91.7	97.1	0.943
Li et al. [17]			100	100	100	-
Sharma and Sharma [12]			95.8	100	97.1	0.841
Song et al. [13]			95.8	100	97.1	0.860
